# Supplementary figures and images for: Temporal Transcriptomic Profiling of the Developing Xenopus laevis Eye
Source: Cells. 2024 Aug 21;13(16):1390. doi: 10.3390/cells13161390 (PMC11352439; doi:10.3390/cells13161390)

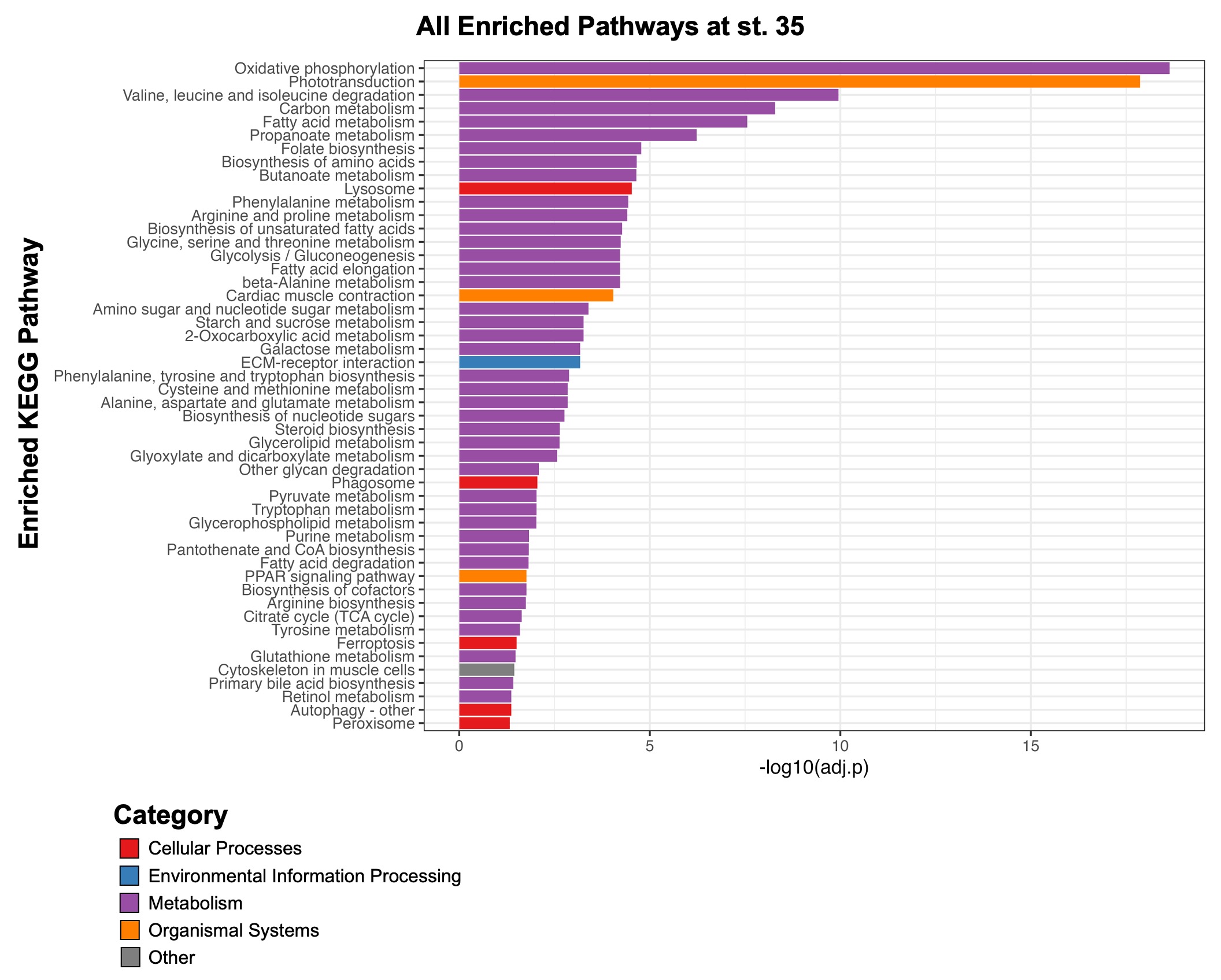

Supplement: Supplementary file 1 [file cells-13-01390-s001.zip › Figure S1.jpg]
